# Dietary supplementation with a wild green oat extract (*Avena sativa* L.) to improve wellness and wellbeing during smoking reduction or cessation: a randomized double-blind controlled study

**DOI:** 10.3389/fnut.2024.1405156

**Published:** 2024-06-19

**Authors:** Marina Friling, Ana María García-Muñoz, Arava Lavie, Silvia Pérez-Piñero, Desirée Victoria-Montesinos, Francisco Javier López-Román, Ana Isabel García-Guillén, Juan Carlos Muñoz-Carrillo, Fernando Cánovas, Eran Ivanir, Jonna Jalanka

**Affiliations:** ^1^IFF Health, Migdal Haemek, Israel; ^2^Faculty of Pharmacy and Nutrition, UCAM Universidad Católica San Antonio de Murcia, Murcia, Spain; ^3^Faculty of Medicine, UCAM Universidad Católica San Antonio de Murcia, Murcia, Spain; ^4^Primary Care Research Group, Biomedical Research Institute of Murcia (IMIB-Arrixaca), Murcia, Spain; ^5^IFF Health & Biosciences, Kantvik, Finland

**Keywords:** tobacco, dietary supplement, Neuravena^®^, quality of life, *Avena sativa*

## Abstract

**Objective:**

Smoking reduction or cessation are critical public health goals, given the well-documented risks of tobacco use to health. Reducing smoking frequency and cessation entirely are challenging due to nicotine addiction and withdrawal symptoms, which can significantly affect mental wellness and overall wellbeing. Previous research has suggested that certain dietary supplements may support smoking cessation and reduction efforts by mitigating these adverse effects. The objective of this study was to assess the effect of supplementation with 900 mg/day of Neuravena^®^, a green oat extract (GOE) of *Avena sativa* L., in enhancing wellness and wellbeing during a smoking reduction or cessation experience.

**Methods:**

This was an 8-week randomized, double-blind, placebo-controlled study, ClinicalTrials Identifier: NCT04749017 (https://classic.clinicaltrials.gov/ct2/show/NCT04749017). Participants were assigned to one of the study groups, 72 participants were assigned to GOE and 73 to placebo. The subjects were followed for 8-weeks intervention period as well as an additional 4-week follow-up period. At subsequent visits, they underwent clinical assessments including assessments of quality of life, perceived stress, depression, nicotine dependence, anxiety, cognitive performance, and specific assessments of craving intensity.

**Results:**

GOE was associated with greater improvements in elements of the abbreviated World Health Organization Quality of Life (WHOQOL-BREF) questionnaire as compared with placebo. Similar results were obtained from the SF-36 questionnaire and a visual QoL analogue scale (VAS). Perceived stress levels showed greater decline from baseline among the GOE supplemented participants as compared to placebo. Sleep quality parameters improved with GOE supplementation and worsened in the placebo group. At the end of the intervention period, the percentage of successful reducers (defined as >20% reduction in daily cigarettes) was higher in the GOE group as compared to placebo (66.7% vs. 49.3%, *p* = 0.034). The improvements from baseline in QoL measures in the GOE group persisted at 4 weeks after termination of the intervention.

**Conclusion:**

GOE supplementation demonstrated greater improvements in quality of life measures, stress and sleep related parameters during a smoking reduction or cessation experience and the product was shown to be safe and well tolerated.

## 1 Introduction

Cigarette smoking is a major public health concern and is a leading cause of disability and premature death (1, 2). Therefore, smoking cessation is recommended due to the many associated advantages for the individual and society, including increasing life expectancy and reduction of health care costs associated with the treatment of smoking related conditions (3). The risk for serious disease is reduced rapidly after smoking cessation regardless of the duration and intensity of previous smoking habit, existing comorbidities, or age of the individual (4, 5).

Despite the clear benefits of smoking reduction, smoking is a very difficult addiction to break (5). Most smokers want to quit, but less than 10% of those who attempt cessation remain abstinent for at least 6 months (6). The mechanism responsible for the addiction has largely been attributed to the pharmacodynamics of nicotine. Nicotine stimulates nicotine acetylcholine receptors (nAChRs) in the brain, which elevate the release of neurotransmitters, mainly dopamine, promoting reward circuits and thus perpetuating consumption (7). Following repeated exposure to nicotine, cessation can also lead to a well characterized withdrawal syndrome that typically includes irritability, anxiety, increased appetite, insomnia, and impaired cognitive performance (8, 9). All these manifestations are temporary, reaching the greatest intensity in the first week and then decreasing over the course of the following 2–4 weeks. More than 40% of smokers report symptoms that persist for longer periods (4, 10).

One of the reasons why people fail to quit smoking is due to the complex interplay between physiological, psychological, and behavioral factors (11–13). Often, smokers report smoking cigarettes to alleviate emotional problems, such as stress relief (14). This is all part of the tobacco withdrawal cycle, misleading the smoker to believe that smoking offers psychological benefits (15). Usually, the level of functional beliefs, such as weight and stress control, associated with smoking correlate with smokers attempt to quit and whether they are successful (14, 16). Moreover, the physiologic effects associated with nicotine withdrawal symptoms may affect one’s overall perception of their quality of life (17). According to the World Health Organization (WHO), health is defined as not merely the absence of disease or infirmity, but a state of complete physical, mental and social wellbeing (18). Indeed, several studies showed that symptoms related to the smoking cessation cause negative alterations in the perceived quality of life (13, 19–21).Therefore, understanding the relationship between one’s perception of overall life satisfaction, may help to improve the individual’s motivation to quit, and enhance relapse prevention strategies (17, 22).

Novel interventions supporting subjects’ wellbeing and quality of life during smoking reduction or cessation experience are necessary. *Avena sativa* (oats) is considered as a nervine herb, supporting the nervous system. It has been used for its physical and psychological effects for centuries, mostly as stress and anxiety reducer, mild anti-depressant, and improving cognitive functions, and is considered to be safe with no known safety concerns at various dosages (23–26). Although some of the oats believed benefits are lacking scientific evidence, studies have showed impact of green oat herb extract (Neuravena^®^, IFF) on mental functions, and maintenance of cardiovascular health (23, 27, 28). Furthermore, Neuravena^®^ has demonstrated an ability to inhibit monoamine oxidase-B (MAO-B) (28, 29). Inhibition of this enzyme increases the dopaminergic availability, and therefore it is hypothesized that it may be beneficial during smoking reduction or cessation by alleviating nicotine withdrawal symptoms and promoting pleasure and a sense of wellbeing (30–33).

Considering the proposed the traditional usage of green oat extract (GOE) and it’s suggested mechanism of action, this study aimed to evaluate the potential effect of supplementing Neuravena^®^ on the wellbeing of smokers during their smoking reduction or cessation experience.

## 2 Materials and methods

### 2.1 Study design and participants

This was a single-center, randomized, double-blind, two-arm parallel-group, placebo-controlled study conducted at the Health Sciences Department of Universidad Católica San Antonio de Murcia (UCAM), in Murcia, Spain between 26th January and 27th July 2021.

Participants were recruited by advertising the study through media, social networks, and e-mail lists of the UCAM University community. Eligible participants were healthy subjects between 18 and 65 years of age, regular smokers of 10 or more cigarettes per day (CPD) at least for the last 6 months, who were willing to reduce/quit daily cigarettes as assessed by the Richmond test (34), who had exhaled carbon monoxide (CO) levels of 10 ppm or more, had a negative urine drug test, and were able to provide informed consent and fully participate in all aspects of the study.

The exclusion criteria were the following: smokers of other nicotine-containing products, such as hookahs, smokeless tobacco or e-cigarettes; use of other smoking cessation aids within the previous 30 days; use of any mineral/vitamin/drug or other supplements within the previous 30 days; presence of any active or chronic disease or chronic medication except for stable antihypertensive and/or antihyperlipidemic agents; presence of depression, anxiety or stress as assessed by psychological evaluation and DASS-21 questionnaire; history of alcohol or drug abuse or dependence within the past year; diagnosis and treatment for mental illness within the past year; known allergy to any of the study components; pregnant or breast-feeding women; and any other laboratory test abnormality, medical condition, or psychiatric disorder that may adversely affect the subjects ability to complete the study according to the investigator’s opinion.

Participants were randomly assigned into the intervention or placebo groups using a computer-generated randomization list with Epidat 4.1 software program (Xunta de Galicia, Santiago de Compostela, Spain) by an independent center. All participants were stratified according to number of daily smoked cigarettes, categorized as below 16 CPD or equal to or greater than 16 CPD, and the willingness to reduce smoking categorized as greater than or equal to a score of 6 and less than a score of 6 (moderate motivation) of the Richmond test (34). A stratified randomization was performed based on two factors (the number of daily smoked cigarettes and the willingness to reduce tobacco smoking). A simple randomization procedure was applied for each of the 4 groups generated, leading to unequal number of subjects between study groups. Restrictive or balanced randomization which requires that the two groups have the same number of subjects, could not be implemented in this study.

The study was conducted in accordance with the Declaration of Helsinki and Good Clinical Practice (GCP) standards (35, 36). The study protocol was approved by the Ethics Committee of Universidad Católica San Antonio de Murcia (code CE102004 approval date October 30, 2020; Murcia, Spain) and registered in ClinicalTrials.gov (NCT04749017). Written informed consent was obtained from all participants.

### 2.2 Intervention and study products

The investigational product consisted of a wild green oat herb extract (Neuravena^®^, IFF España, Sant Cugat del Vallés, Barcelona, Spain). The daily dosage consisted of two capsules each containing 450 mg of Neuravena^®^, or 519 mg maltodextrin as placebo. Both GOE and placebo products were encapsulated by an independent company (Laboratorios Admira, Murcia, Spain), providing an identical hard gelatin capsule format. All participants were instructed to take two capsules daily (one in the morning and one in the afternoon) for 8 consecutive weeks (60 days). The GOE dose of 900 mg/day was selected as it was shown to be safe in previous clinical studies (20, 21, 30).

### 2.3 Study procedures and data collection

**Figure 1:**
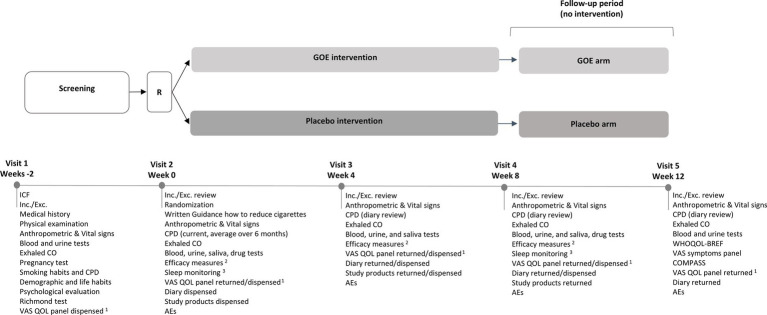
Study design. ^1^VAS QOL panel was completed every 2 weeks the night prior to the next visit or during weeks between visits; ^2^Efficacy measures: WHOQOL-BREF, SF-36, VAS symptoms panel, PSS, STAI, BDI-II, MNWS, FTND, COMPASS, Cue induced craving test (QSU + VAS); ^3^Sleep was monitored over 3 working days +1 weekend before visit 2 and visit 4 (8 weeks); R, Randomization; ICF, Informed Consent Form; Inc./Exc., Inclusion/Exclusion; CPD, Cigarettes Per Day; CO, Carbon Monoxide; VAS, Visual Analogue Scale; QOL, Quality of Life; AEs, Adverse Events.

The study design and procedures taken during the trial are visualized in Figure 1. Each subject participated in an 8-week intervention period and an additional 4 week follow up period. This 12-week study period included a baseline visit (Visit 2), mid-study visit at 4 weeks (Visit 3), end of intervention visit at 8 weeks (Visit 4), and last visit at 12 weeks (Visit 5).

The screening visit (Visit 1) took place 15 days prior to the baseline visit, where the inclusion and exclusion criteria were evaluated, and the written informed consent was obtained. Once consent was obtained, participants completed a medical history and physical examination. Vital signs, height, weight, blood, and urine samples (including pregnancy test for women), as well as exhaled carbon monoxide (CO) were collected. Participants were evaluated for their psychological eligibility (depression, anxiety, and stress levels) by completing the DASS-21 questionnaire and a psychologist’s evaluation. Participants’ willingness to reduce daily cigarette number was assessed by the Richmond test. Furthermore, personal, demographic and life habitual information was collected: Age, ethnicity, familiar status, education, social habits, smoking habits (past/current), etc. Eligible participants were randomized and allocated into one of the groups at the baseline visit (Visit 2). Thereafter, the clinical assessments started and were similar on all intervention visits (Visit 2–Visit 4).

At Visits 2–4, participants were instructed to consume a light lunch 2 h before the visit and were requested to abstain from smoking once entering the clinic. The timeline and sequence of assessments is provided in Table 1. All questionnaires and scales were adapted and validated for Spanish. On each visit, eligibility criteria were checked, and vital signs and body weight were recorded. Participants completed the following efficacy assessments: the abbreviated World Health Organization Quality of Life (WHOQOL-BREF)^1^ (37); the Short-Form Health Survey (SF-36) (38); the Perceived Stress Scale (PSS) (39); the Beck Depression Inventory-II (BDI-II) (40); the Minnesota Nicotine Withdrawal Scale (MNWS) (41); the Fagerström Test for Nicotine Dependence (FTND) (42); the State–Trait Anxiety Inventory (STAI) (43); a cognitive battery using the Computerized Mental Performance Assessment System (COMPASS, Northumbria University, UK, instruction screens adapted for Spanish); and a 100 mm visual analogue scale (VAS) symptoms panel developed for this study. Craving intensity was assessed twice, before and after a provocation procedure. After participants abstained smoking for ~150 min, craving assessment was conducted in a cue induced test. Craving was provoked by participants’ exposure to smoking related cues which included visual stimuli of seven categories (social celebration, study environment, smokers, coffee and cigarette, cigarette and pack, free time, ashtray, and cigarettes) and presence of tobacco as a tactile and olfactory stimulus. The level of craving was assessed before and after the provocation by (1) Brief Questionnaire on Smoking Urge questionnaire (QSU-Brief) (44). (2) A 100 mm VAS craving question developed for this study.

**Table 1:** Timeline and sequence of assessments.

Time from visit start	Procedure and assessment
Evening before	VAS QoL panel (diary)
−120 min	Consumed light lunch (at home)
−60 min	Product consumption (at home)^1^
0–45 min	Abstinence from smoking started when attending the clinic
	Inclusion/exclusion criteria and diary review
	Vital signs (heart rate, blood pressure, and temperature), and weight
	WHOQOL-BREF
	SF-36^2^, PSS^2^, BDI-II^2^
	Exhaled CO (20–30 min post abstinence)
	Blood (cotinine), urine (cotinine, biopyrrin^2^, drug test^3^), and saliva (cortisol^2^) samples collection
	VAS symptoms panel
45–90 min	Break
90–150 min	COMPASS cognitive test panel^4^
	MNWS^2^, FTND^2^, STAI^2^
	Pre exposure to cue assessment (QSU-Brief, VAS craving level)^2^
	Exposure to cues provoking craving^2^
	Post exposure to cue assessment (QSU-Brief, VAS craving level)^2^
	Saliva (cortisol) sample collection^2^

^1^Product consumption applies only for visits 3 and 4. ^2^Assessments apply only for visits 2–4, not performed at follow-up. ^3^Drug test applies to visits 2 and 4 only, including THC, amphetamines, methamphetamines, cocaine, and opioids. ^4^On visit 5, performed within 50–75 min; Min, minutes.

Perceived QoL was evaluated by a 100 mm VAS QoL panel developed for this study and recorded by participants in their diary every 2 weeks (from the night before Visit 2 to the night before Visit 5). In addition, sleep quality was assessed in all participants by using a wrist-worn accelerometer during 3 consecutive weekdays and 1 weekend day before Visit 2 and Visit 4. A more detailed explanation of all assessments, scores and necessity of use can be found in Supplementary Table S1.

Biochemical measurements related to smoking were exhaled CO level (9 ppm was defined as a cutoff point to identify current smokers) (7) and blood and urine cotinine. Urinary and saliva cortisol levels were analyzed for stress related biochemical measurements. Urinary drug test was performed (on Visit 2 and Visit 4 only) for tetrahydrocannabinol (THC), amphetamines, methamphetamines, cocaine, and opioids. More details on biochemical measurements can be found in Supplementary Table S2.

After the 8-week intervention, participants were followed for an additional 4 weeks. At the end of the follow-up period (Visit 5), participants completed the WHOQOL-BREF questionnaire and the COMPASS tests, exhaled CO was measured, blood and urine samples for cotinine levels were collected, and VAS symptoms panel was completed.

At the end of Visits 2 and 3, participants received the exact number of capsules required for the next 4 weeks of intervention, a diary for daily record of CPD and completion of VAS QoL panel was provided. Subjects were requested to return all blisters they have received in the previous visit, and compliance was monitored at visits 3 and 4 by counting the number of capsules remaining in the blisters pack. Dose compliance was defined as the number of capsules taken by a participant during 4 weeks of the study period divided by the number of capsules expected to be taken during these 4 weeks multiplied by 100. Compliance was assessed for consumption during the first and second 4 weeks period, and during the overall study period (throughout 8 weeks). In addition, the sleep accelerometer was dispensed to participants a few days before Visits 2 and 4 to evaluate the quality of sleep. A diary to record CPD was dispensed at the end of Visit 4. Adverse Events (AE) were recorded on Visits 2–5 for safety evaluation.

### 2.4 Study endpoints

The primary end points of the study were significant differences between the GOE and placebo group in the change in WHOQOL-BREF from baseline to the end of the trial (8 weeks).

The secondary endpoints were the following: differences between the groups in changes from baseline of QoL as measured by WHOQOL-BREF at 4 weeks and to the end of the follow-up period (Visit 5); differences between the groups in changes from baseline to weeks 4 and 8, and during the follow-up period in the collected questionnaires: perceived QoL (measured by VAS QoL panel); severity of physical symptoms related to the smoking reduction or cessation experience and impact on ability to function (measured by VAS symptom panel); frequency of physical symptoms related to the smoking reduction or cessation experience; extent of smoking reduction (averaged CPD across 7 days prior to the respective visit and biochemical markers: exhaled CO, cotinine level in both blood and urine); proportion of participants reporting 7-day smoking abstinence (verified by exhaled CO readings of <9 ppm); and cognitive performance (measured by COMPASS). Additionally, differences between the groups in changes from baseline to weeks 4 and 8 were evaluated in: general health aspects (assessed by SF-36); perceived stress (assessed by PSS); stress level (measured by salivary cortisol and urinary biopyrrin); anxiety level (assessed by STAI); depression level (assessed by BDI-II); withdrawal symptoms appearance (assessed by MNWS); nicotine dependency levels (assessed by FTND); urge for smoking following cue-induced craving test (assessed by QSU-brief questionnaire and VAS); stress levels following cue-induced craving test (measured by salivary cortisol); and sleep quality (measured by sleep accelerometer; change from baseline to week 8 only). Safety endpoints were anthropometric variables, vital signs, and AEs.

Exploratory endpoints included within group changes in the measured variables from baseline to weeks 4 and 8 and during follow-up.

### 2.5 Statistical analysis

A sample size of 140 participants, with a total of 160 participants when assuming a dropout rate of 12.5%, was estimated to reflect a difference between the groups with an effect size of 0.48 and 80% power using an independent *t*-test with 0.05 two-sided statistically significant level. The intent-to-treat (ITT) dataset included all randomized participants for whom any post-randomization efficacy evaluation was available. The per-protocol population (PP) included all subjects in the ITT population who completed post-randomization evaluations at all study visits and did not have any major protocol violations. The main evaluations of the efficacy parameters were based on the ITT population. Linear mixed model for repeated measures (MMRM) was fitted for the primary endpoint analyzing the change from baseline to week 8 between the study groups. The model included fixed effects for baseline (week 0), week number (week 8), and product group (GOE and placebo). Study subjects were considered as random effects. For secondary and exploratory endpoints, an independent *t*-test was used for continuous variables. For continues variables with a frequency of occurrence of less than 30 subjects with non-normal distribution, the non-parametric Mann–Whitney U test was used to compare the mean rank of two not related samples and determine differences. For categorical variables, the chi-square test was used. In addition, comparison was conducted within each group between respective time points. Paired *t*-test was used for continuous variables, and McNemar’s test was used for categorical variables. Analysis was corrected for baseline values as appropriate. Statistical significance was set at *p* < 0.05. Statistical analyses were performed with SPSS 25.0 (or higher) for Windows.

## 3 Results

### 3.1 Study population

**Figure 2:**
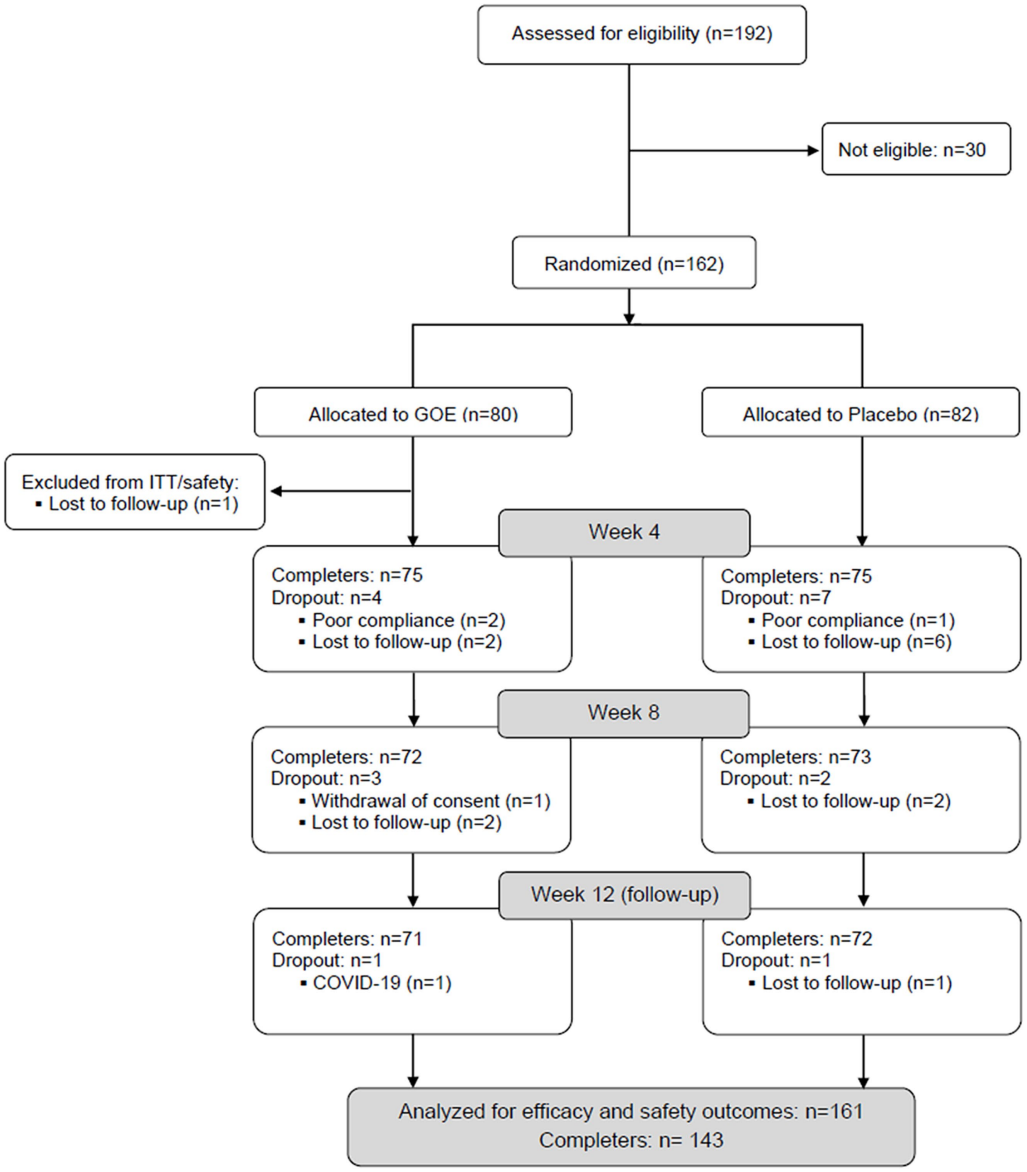
Flow chart of the study population (GOE: green oat extract).

A total of 192 participants were assessed for eligibility and 162 were randomized, 80 were assigned to the GOE group and 82 to the placebo group. One participant randomized to the GOE group discontinued the study during the initial visits and, therefore, was not included in the further analysis. Finally, 71 participants from the GOE group and 72 from the placebo group completed the five study visits. The flow chart distribution of participants is shown in Figure 2.

There were 74 men and 87 women (54%) with a mean age of 29.59 (±10.76) years participating in the study. The participants demographics are shown in Table 2. The mean age when participants started smoking was 16.12 (±3.32) years. Participants smoked 13.47 (±10.63) years on average. The mean CPD number was 16.24 (±5.46), and the motivation to quit was assessed to be moderate (Richmond score 6.29 ± 1.7). No significant differences were found between the participants assigned to the GOE group and placebo group, except for participants going out more frequently in the GOE group.

**Table 2:** Demographic and clinical characteristics of participants randomized into the study.

Variables	Total (*n* = 161)	Study arm		*P*-value^1^
		GOE (*n* = 79)	Placebo (*n* = 82)	
Age, years	29.6 (10.8)	28.3 (10.4)	30.8 (11.1)	0.133
**Sex, n (%)**				
Male	74 (46.9)	41 (51.9)	33 (40.2)	0.138^2^
Female	87 (54.0)	38 (48.1)	49 (59.8)	
Body mass index, kg/m^2^	25 (4.8)	24.8 (4.9)	25.1 (4.7)	0.703
**Ethnicity, n (%)**				
Caucasian	159 (98.8)	79 (100.0)	80 (97.6)	0.377^2^
Arab	1 (0.6)	0	1 (1.2)	
White Hispanic	1 (0.6)	0	1 (1.2)	
**Marital status, n (%)**				
Married/living with partner	42 (26.1)	20 (25.3)	22 (26.8)	0.089^2^
Single	108 (67.1)	57 (72.2)	51 (62.2)	
Divorced/separated	11 (6.8)	2 (2.5)	9 (11.0)	
**Social habits, n (%)**				
Going out <3 times a week	118 (73.3)	50 (63.3)	68 (82.9)	0.005^2^
Going out >3 times a week	43 (26.7)	29 (36.7)	14 (17.1)	
Age started daily smoking, years	16.1 (3.3)	16.4 (3.4)	15.9 (3.3)	0.351
Total smoking period, years	13.5 (10.6)	11.9 (10.3)	15 (10.8)	0.070
Smokers with quitting attempts, n (%)	83 (51.6)	36 (45.6)	47 (57.3)	0.136 ^2^
Cessation period, months	9.8 (15.3)	6.5 (8.0)	12.3 (18.9)	0.080
Smokers with attempts to reduce smoking, n (%)	85 (52.8)	39 (49.4)	46 (56.1)	0.392 ^2^
Reduced number of cigarettes	5.8 (3.6)	5.3 (3.5)	6.3 (3.7)	0.236
DASS-21 total score	10.1 (5.9)	10.5 (5.9)	9.8 (6.0)	0.448
Cigarettes per day	16.2 (5.5)	15.7 (5.0)	16.7 (5.9)	0.231
Richmond test score	6.3 (1.7)	6.1 (1.6)	6.5 (1.8)	0.179

Data expressed as means (standard deviation) unless otherwise stated. DASS-21: Depression Anxiety and Stress Scale 21. ^1^*p*-values were derived from an independent *t*-test. ^2^*p*-values were derived from a Chi-square test.

### 3.2 Primary outcome- changes in quality of life from baseline to the end of the intervention assessed by WHOQOL-BREF

**Figure 3:**
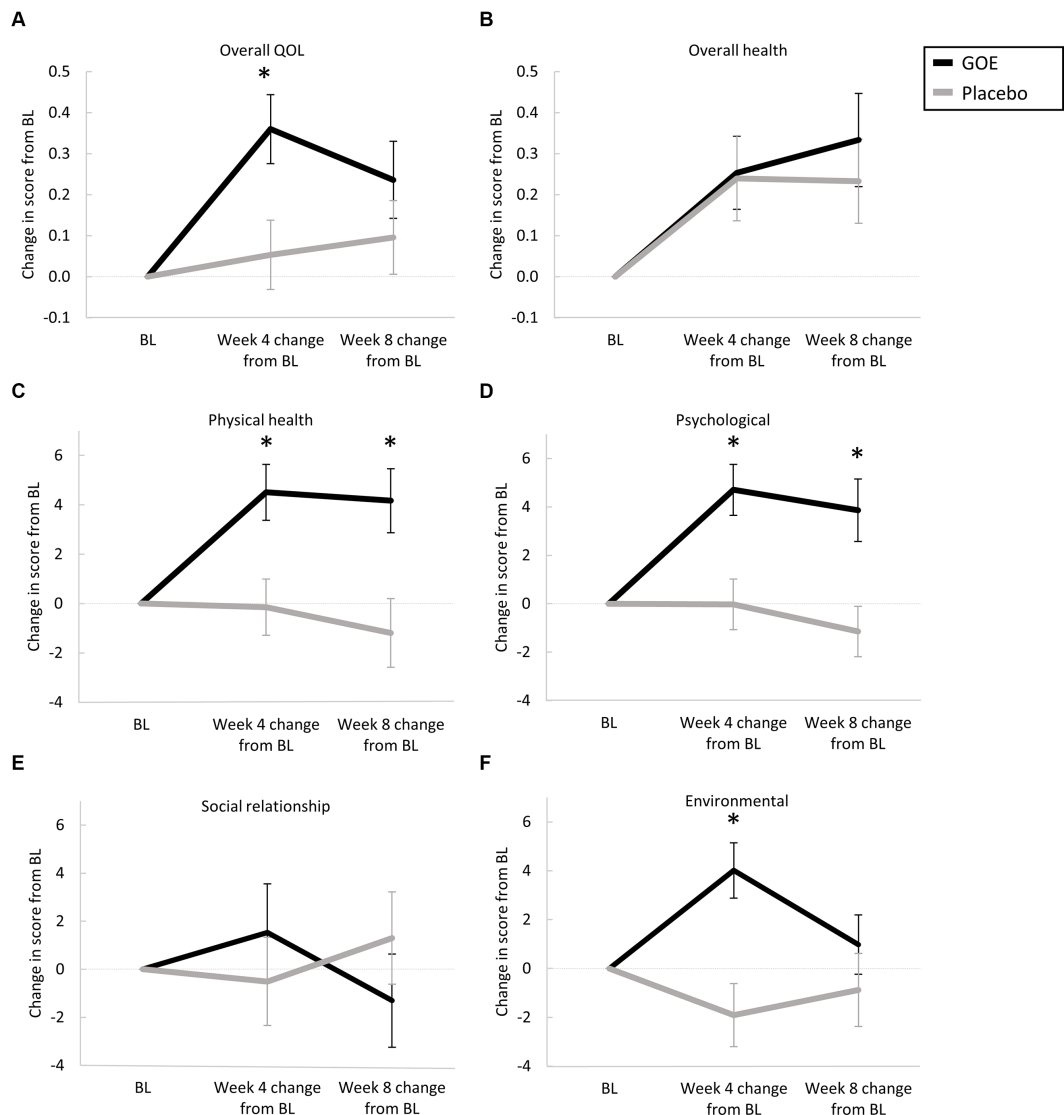
Changes in QoL parameters at baseline and following 8 weeks. **(A)** Overall QoL. **(B)** Overall health. **(C)** Physical health. **(D)** Psychological. **(E)** Social relationships. **(F)** Environmental. *p < 0.05 for between-group comparisons.

The primary endpoint of the study was differences between study groups in the changes in QoL scores assessed by WHOQOL-BREF from baseline to 8 weeks. There was a statistically significant improvement from baseline to week 8 in physical health and psychological domains for the GOE group as compared to placebo (*p* = 0.006 and *p* = 0.008, respectively; Table 3; Figure 3). There were no significant differences in the other measured points of the questionnaire.

**Table 3:** Quality of life parameters at baseline and change following 8 weeks.

WHOQOL-BREF Item/domain	GOE group		Placebo group		*p*-value
	Baseline (*n* = 79)	Change from baseline to week 8 (*n* = 72)	Baseline (*n* = 83)	Change from baseline to week 8 (*n* = 73)	
Overall QoL	3.6 (0.9)	0.2 (0.8)*	3.8 (0.7)	0.1 (0.8)	0.282
Overall health	3.2 (0.9)	0.3 (1.0)*	3.2 (0.9)	0.2 (0.9)*	0.512
Physical health^1^	72.5 (13.4)	4.2 (11.0)*	74.8 (12.8)	−1.2 (11.8)	**0.006**
Psychological^1^	64.5 (15.4)	3.9 (10.9)*	69.6 (14.5)	−1.2 (8.9)	**0.008** ^†^
Social relationships^1^	70.3 (18.7)	−1.3 (16.4)	69.2 (19.3)	1.3 (16.4)	0.341
Environmental^1^	68.0 (11.5)	1.0 (10.3)	69.7 (14.2)	−0.9 (12.8)	0.339

WHOQOL-BREF: abbreviated World Health Organization Quality of Life. A higher score indicated better outcome. ^1^Domains raw score were transformed on a scale from 0 to 100; Data is expressed as mean (standard deviation); *P*-values are based on MMRM analysis for the change from baseline to week 8 between the groups; ^†^Based on analysis of covariance controlled for baseline; *Significance based on paired *t*-test between baseline to a respective time point; Bold indicates significant differences at *p* < 0.05.

### 3.3 Secondary outcomes and exploratory analysis

#### 3.3.1 Quality of life and general health aspects

Changes in the QoL parameters measured by the WHOQOL-BREFF from baseline to the mid-point of the intervention (week 4), showed significant improvements for the GOE group in overall QoL, physical health, psychological, and environmental as compared to placebo (Table 4; Figure 3). Over time, significant improvements within the GOE group were observed in all parameters except social relationship (Supplementary Table S3).

**Table 4:** Descriptive parameters of the variables studied with statistically significant differences.

Measurement	Item	GOE			Placebo			*p*-value		
		Baseline	Change from baseline		Baseline	Change from baseline		P1	P2	P3
			Week 4	Week 8		Week 4	Week 8			
		*N* = 79	*N* = 75	*N* = 72	*N* = 82	*N* = 75	*N* = 73			
WHOQOL-BREF (quality of life)	Overall QoL	3.6 (0.9)	0.4 (0.7)	0.2 (0.8)	3.8 (0.7)	0.05 (0.7)	0.1 (0.8)	0.077	**0.011**	**0.282**
	Physical health	72.5 (13.4)	4.5 (9.9)	4.2 (11.0)	74.8 (12.8)	−0.2 (9.8)	−1.2 (11.8)	0.258	**0.004**	**0.006**
	Psychological	64.5 (15.4)	4.7 (9.1)	3.9 (10.9)	69.6 (14.5)	−0.0 (9.1)	−1.2 (8.9)	**0.033**	**0.009** ^**†**^	**0.008** ^†^
	Environmental	67.9 (11.5)	4.0 (9.8)	1.0 (10.3)	69.7 (14.2)	−1.9 (11.2)	−0.9 (12.8)	0.387	**0.001**	0.339
VAS QoL panel (perceived quality of life)	Confidence level (cm)	6.7 (2.1)	0.5 (2.1)	0.4 (2.3)	7.3 (2.0)	−0.4 (1.7)	−0.2 (1.6)	0.061	**0.006**	**0.047**
SF-36 (general health aspects)	Bodily pain	76.6 (20.2)	4.8 (21.0)	5.6 (25.0)	79.3 (20.6)	−2.9 (22.9)	5.1 (19.0)	0.405	**0.032**	0.892
	Vitality	61.6 (15.3)	3.6 (15.2)	7.8 (15.8)	65.2 (13.9)	0.4 (13.6)	1.0 (15.3)	0.118	0.179	**0.009**
	Mental health	70.4 (15.2)	2.7 (12.7)	5.5 (13.2)	74.8 (15.1)	0.1 (13.7)	0.8 (15.2)	0.069	0.229	**0.048**
PSS (perceived stress)	PSS score	21.0 (8.2)	−2.1 (5.7)	−3.2 (6.5)	19.1 (7.6)	−0.6 (6.3)	−0.6 (7.5)	0.124	0.111	**0.028**
Sleep quality	WASO (min)	38.0 (18.1)	–	−0.7 (16.9)	35.7 (16.5)	–	5.4 (19.0)	0.403	–	**0.047**
Successfully reduced smoking, n (%)		–	42 (54.5%)	48 (66.7%)	–	33 (43.4%)	36 (49.3%)	–	0.169*	0.034*

GOE, Green Oat Extract; WHOQOL-BREF, abbreviated World Health Organization Quality of Life; VAS, Visual Analogue Scale; F-36, Short Form-36 Health Survey; PSS, Perceived Stress; COMPASS, Computerized Mental Performance Assessment System; Data is expressed as mean (standard deviation); Data is expressed as mean (standard deviation) unless otherwise stated; P1, Comparison between baseline values; P2, Comparison between GOE and placebo for change from baseline to week 4; P3, Comparison between GOE and placebo for change from baseline to week 8; *P*-values are based on an independent *t*-test; ^†^Based on analysis of covariance controlled for baseline; *P-values are based on the Chi-squared test; Bold indicates significant differences at *p* < 0.05.

The VAS QoL panel showed significant improvement for the GOE group in the perceived confidence level as compared to placebo following 4 and 8 weeks of intervention (Table 4). Significant improvements in the GOE group over time as compared to the baseline occurred in most of the measured variables as shown in Supplementary Table S4. The placebo group showed a significant improvement in sleep quality, decrease in the urge to smoke, and a decline in confidence level as compared to baseline (Supplementary Table S4).

Assessment of general health with the SF-36 questionnaire showed a significant improvement in the vitality and mental health domains following 8 weeks of GOE intervention as compared to placebo. A significant improvement in the change in bodily pain domain was shown following 4 weeks intervention of GOE as compared to placebo (Table 4). In addition, the change in the physical component summary score showed a significant increase for the GOE group compared to placebo. Overtime, a significant improvement from baseline was observed within the GOE group in physical functioning, physical role limitations, bodily pain, vitality, mental health, physical health component summary, and mental component summary. On the other hand, the placebo group showed significant improvement in bodily pain only as compared to the baseline (Supplementary Table S5).

#### 3.3.2 Smoking behavior

**Figure 4:**
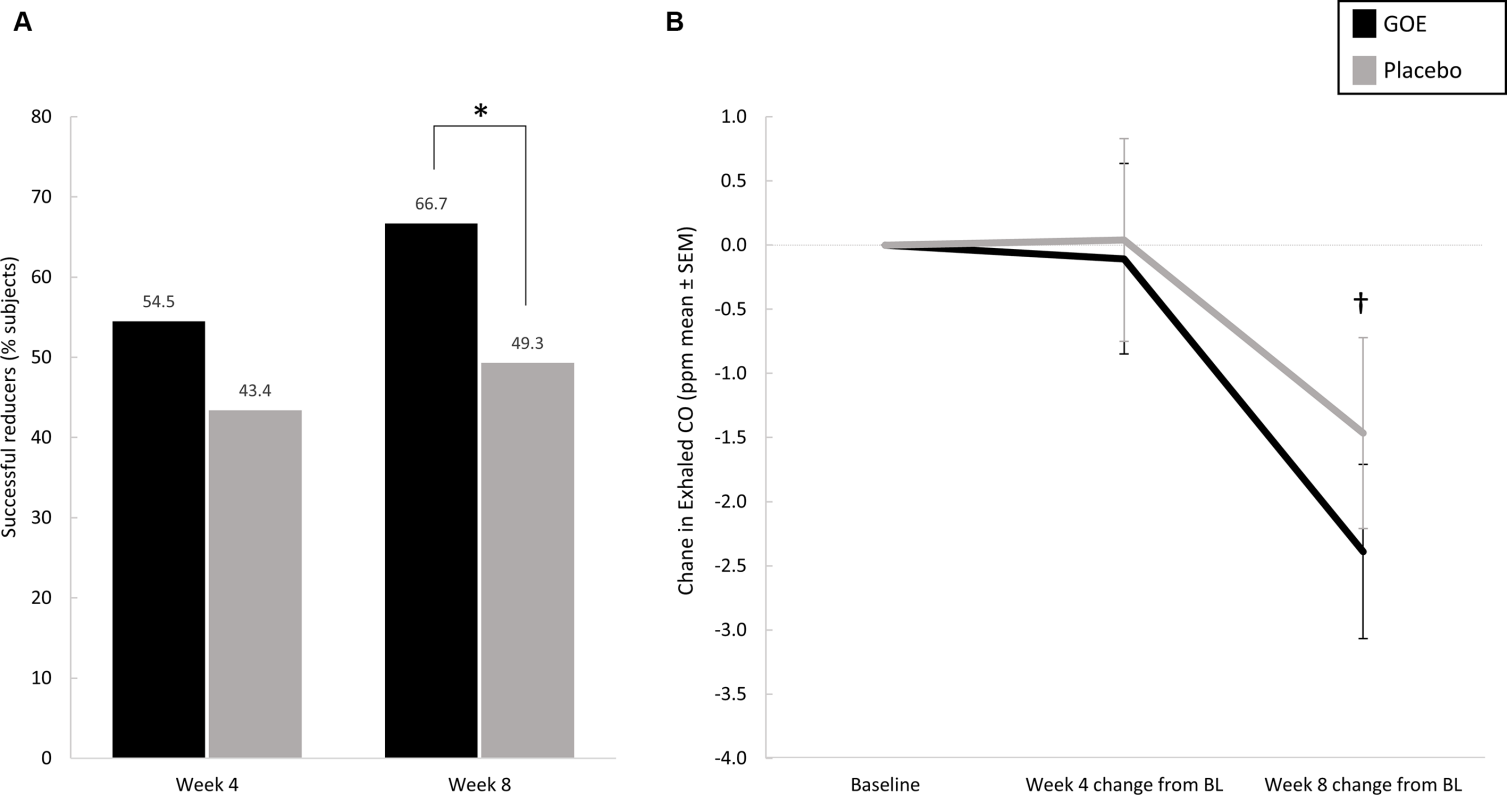
**(A)** Percentages of successful reducers (20% or more reduction in CPD) in the two study groups, with significant differences in favor of the GOE group at week 8. **(B)** Exhaled CO measurements (ppm) in the two study groups. *p < 0.05 for between-group comparisons. ^†^*p* < 0.05 for change at week 8 compared to baseline in the GOE group. BL, baseline.

Both groups showed a significant reduction in daily cigarette consumption (CPD) over time from baseline throughout the study period. However, significant differences in CPD between the treatment groups was not observed following 4 and 8 weeks of intervention (Supplementary Table S6). Following 8 weeks of intervention, a significantly higher proportion of participants were considered successful reducers (CPD reduction of >20%) in the GOE group as compared to placebo (66.7% vs. 49.3%; Table 4; Figure 4A). As an objective measurement of cigarette consumption reduction, we found that the exhaled CO levels were significantly decreased after 8 weeks as compared to baseline only in the GOE group (Table 4; Figure 4B). With respect to blood or urinary cotinine levels, significant differences were not found between study groups, nor in each group relative to baseline values (Supplementary Table S6). Analysis of participants who abstained from cigarettes did not show any significant results as only one participant in each group successfully abstained from cigarettes at 8 weeks (Supplementary Table S6).

#### 3.3.3 Stress levels

The perceived stress levels as measured with the PSS scale declined in both groups over the 8 weeks of intervention, with a significantly greater decrease in the GOE group as compared to placebo (Table 4). Moreover, the perceived stress score significantly reduced overtime (week 4 and 8) as compared to baseline only in the GOE group (Supplementary Table S7). No significant reduction of stress levels was observed with in the placebo group.

Stress-related biomarkers (urinary biopyrrin and salivary cortisol) did not differ between the study groups and a similar significant increase in urinary biopyrrin was observed in both study groups compared to baseline values (within-group differences, *p* < 0.05).

#### 3.3.4 Physical and psychological symptoms related to the smoking reduction or cessation experience

As measured with the VAS symptom panel at the initial visit, a higher percentage of participants reported headaches in the GOE group vs. placebo, but the difference was not statistically significant (*p* = 0.187) (Supplementary Table S8). However, there was a significant decrease in reported headache in both groups after 8 weeks of treatment as compared to baseline. The percentage of participants who reported to experience any symptom at baseline was 74.7 and 62.2% within the GOE and placebo groups, respectively (Supplementary Table S8). Following the 8 weeks of intervention, the percentage of participants experiencing any symptom significantly decreased within both study groups to 55.6 and 39.7%, respectively. The percentage of participants who experienced any symptom, and participants who reported an impact on the ability to function were comparable between study groups at both time points. Following 8 weeks of intervention (within group comparison), both products resulted in significantly fewer participants reporting any symptoms, coughing, sore throat, and headache. Additionally, the placebo group resulted in less participants who reported tingling in hands and feet, sweating, and dizziness/vertigo over time. In comparison of symptoms severity, no significant differences were observed between the groups or overtime in any of the symptoms following 4 and 8 weeks of consumption (Supplementary Table S8).

#### 3.3.5 Depression and anxiety levels

There were no significant differences between study groups in reported anxiety levels (STAI questionnaire) or depression (BDI-II) levels. Study groups presented a decrease in subscales scores overtime, however only the STAI-Trait (general anxiety feeling) score was found to be significantly different compared to baseline in both groups (Supplementary Table S9). BDI-II scores within the GOE group decreased significantly following 4 and 8 weeks as compared to baseline. In the placebo group, a significant decrease was observed only following 4 weeks of consumption (Supplementary Table S9).

#### 3.3.6 Craving level, nicotine dependency, and withdrawal symptoms

No significant differences were observed between the study groups in craving parameters, nicotine dependency and withdrawal symptoms. Within both study groups, there was a significant decline in some of the craving parameters, cigarette dependency and withdrawal test measures at weeks 4 and 8 when compared to baseline values (Supplementary Table S10).

#### 3.3.7 Sleep quality

At 8 weeks, a significant improvement in the wakefulness after sleep onset (WASO) was recorded in the GOE group as compared to the placebo group (Table 4). Furthermore, sleep efficiency, WASO, and time of mean wake episode were also deteriorated in the placebo group overtime, while there were no significant changes in the GOE group (Supplementary Table S11).

#### 3.3.8 Cognitive performance

We did not observe significant differences in the cognitive performance between the study groups. The results of cognitive performances using the COMPASS platform are summarized in Supplementary Table S12. Both groups showed significant improvements in some parameters, and worsening in others as shown in Supplementary Table S12, therefore the cognitive results were considered inconclusive and not clinically meaningful.

#### 3.3.9 Follow-up period

After the 8-weeks intervention period, participants went through an additional 4 weeks of follow-up (no intervention) to obtain information on differences in their QoL parameters, physical symptoms, cognitive performance, and smoking behavior. After the 4-week follow-up period the GOE group showed a significant improvement as compared to placebo in the overall QoL and social relationship domains of the WHOQOL-BREF (Supplementary Table S3). The GOE group also showed improvement in social relationship overtime. Furthermore, significant differences were found in the level of concentration/focus as measured by the VAS QoL panel, where the GOE group showed improvement as compared to placebo. Significant differences overtime were observed at week 12 in the concentration/focus level records for the GOE group, and an increase in physical health, happiness, satisfaction with leisure time activity and mood level in the placebo group (Supplementary Table S4).

No significant changes in CPD, exhaled CO, urinary or blood cotinine levels between the two study groups were found from weeks 8 to 12. Abstinence after the follow-up period was achieved in 2 participants in the GOE group and 3 participants in the placebo group (Supplementary Table S6). Results of cognitive performance after the follow up period are shown in Supplementary Table S12. Like the cognitive outcomes in the intervention period, both groups showed significant improvements in some parameters, and worsening in others and the results are inconclusive.

#### 3.3.10 Adverse events and compliance

A total of 35 AEs occurred among the 79 participants in the GOE group, and 48 AEs were reported among 82 participants in the placebo group. A majority of the complaints were related to musculoskeletal and gastrointestinal AEs, particularly muscle pain, back pain, and abdominal discomfort. All AEs were of mild intensity and unrelated to the study products, except for two cases of loss of appetite (GOE and placebo groups, one participant in each) and one case of gastroenteritis in the placebo group, which were considered possibly related with the study product (data not shown).

The overall mean compliance with the study products was 97.5% (± 3.1) with no significant difference between the study groups (detailed in Supplementary Table S10).

## 4 Discussion

This study evaluated the potential effects of GOE on wellness and wellbeing of healthy individuals during their smoking reduction or cessation experience. GOE has been shown to act as MAO-B inhibitor and could potentially increase dopaminergic availability. This is the hypothesized mechanism contributing to reduction of physical and psychological symptoms associated with smoking reduction or cessation. This study demonstrated that 8-week consumption of GOE during smoking reduction or cessation experience was associated with greater improvements from baseline in physical health and psychological domains (WHOQOL-BREF, primary outcome). Similarly, additional measured aspects of quality of life, physical and mental subscales of general health, perceived stress and the quality of sleep have also showed improvement with GOE supplementation as compared to placebo and across the intervention period, when compared to baseline.

Participants in this study could be classified as moderate nicotine dependents based on their reported CPD and FTND scores, this classification also stands in regard to the measured QOL scores in WHOQOL-BREF domains which are considered appropriate for mild to moderate dependence, as was observed in other studies (45, 46). In general terms, quality of life is associated with person’s total wellbeing, psychological, social, and physical health status and the interrelations between these aspects (18, 46, 47). The physical aspects of quality of life are likely related to a subjects’ general functioning, disabilities or impairments which distress the perception of their health, while the psychological and mental parameters are commonly associated with depression, anxiety, and stress, which are common among smokers (22, 48). This study demonstrated that GOE supported the participants’ physical and psychological aspects of quality of life, therefore enabling a more successful reduction experience. This was observed by the significant difference of those subjects who successfully reduced smoking.

Many smokers are under the impression that smoking aids in their ability to manage stress, and they fear that quitting smoking would lead to the loss of an efficacious stress-coping mechanism. However, there is strong evidence that the act of quitting smoking is linked to a reduction in stress levels (49, 50). Others suggest that the interplay between smoking and stress is bidirectional, wherein unsuccessful cessation or relapse could heighten an individual’s stress levels, while conversely, stress might interfere with smoking cessation success (51). In light of this conceptual framework, the consumption of GOE has demonstrated the ability to reduce stress levels among individuals trying to reduce or cease smoking. Consequently, this intervention holds the potential to foster a more constructive and positive experience.

An additional factor that impacts the health and wellbeing of any individual, particularly smokers, is sleep quality. Inadequate sleep can lead to a wide range of disorders (52). In our study, sleep quality among placebo consumers was significantly worsened across study period with poorer sleep efficiency and more wakefulness time, while GOE consumers maintained their sleep quality and experienced significantly less minutes of wakefulness after sleep onset. In light of other studies suggesting that targeting sleep quality could be a potential treatment for relapse prevention (53), GOE is potentially supporting smokers experience to reduce and quit smoking also by maintaining their sleep quality.

We showed that subjects consuming GOE for 8 weeks included significantly more successful reducers (>20% reduction in CPD) as compared to the placebo group. This finding reinforces the suggested association between improvement in subjects’ wellness and wellbeing to successful reduction experience. The negative relationship between smoking and quality of life and the association with number of cigarettes smoked is well known and demonstrated in several previous studies (22, 47, 54). Smoking reduction is often suggested as a step toward quitting for individuals who are unable or not willing to quit smoking abruptly. Studies have shown that smoking reduction increases the probability of cessation in the long term, as gradual and controlled reduction is related with less withdrawal symptoms and a success feeling that may motivate smokers to quit (55–57). Therefore, improvement in an individual’s wellness and wellbeing during the experience of reduction could also play an important role contributing to cessation success. Although the exhaled CO levels were significantly reduced from baseline values in the GOE group, there were no significant differences between the groups for the change in exhaled CO level. This could be related to the relatively small decrease and accuracy of the method or to the relatively high variance between the participants in both groups.

In relation to physical and physiological symptoms, the consumption of GOE did not seem to have any impact on the severity or frequency of symptoms related to the smoking reduction or cessation experience, impact on general functioning, nicotine withdrawal symptoms, nicotine dependence level, anxiety, depression, urge to smoke, as well as the related biomarkers. Although there was a significant difference in proportion of participants reporting headache, which appeared to be higher in the GOE group as compared to placebo, this difference was not considered a clinically meaningful in terms of tolerability outcome, as both groups showed significant reduction overtime in the reported frequency. Similarly, reward craving score and momentary urge to smoke following cue exposure test were significantly different at week 4 only with better outcome for placebo, but this was also not considered clinically meaningful. It was previously demonstrated that GOE had improved cognitive performance (28), however, cognitive performance results of the present study were inconclusive. This may be associated with the smoking abstinence time before participants conducted the test (>1.5 h). It was suggested in other studies, that nicotine has a temporary effect in enhancing cognitive performance. Therefore, during smoking deprivation, cognitive deficits are observed in participants who are deprived smoking than those who actively smoked immediately before the test or nonsmokers. Research also showed that smoking is significantly associated with cognitive deficits regardless of depravation time, and more smoking tend to show larger deficits (58–61).

During the follow-up period, smoking behavior remained consistent with the end of intervention period. Participants in the GOE arm reported significant improvement in several quality-of-life parameters including overall QoL, social relationship, and concentration/focus level as compared to placebo. These differences may be attributed to the higher numbers of successful smoking reducers that were observed at the end of the intervention period following GOE consumption and were consistent during the follow up period. Coughing was improved in favor of the placebo group, but this difference is not considered clinically relevant. In relation to cognitive performance, tests results remained inconclusive, similar to the intervention period. In terms of safety, GOE was well tolerated by the study participants, all AEs were mild and only one was considered possibly related to study product.

### 4.1 Limitations and strengths

Despite the asset of this randomized, double-blind, placebo-controlled design there were limitations to the study, one of which being the relatively short intervention period not showing a long-term effect of the product. Additionally, the studied population mostly consisted of mild to moderate tobacco dependent participants, therefore not showing efficacy to those with high tobacco dependency. Nevertheless, this study is considered clinically relevant and showed adequacy in assessing QoL during smoking reduction or cessation experience while demonstrating outcome measures in line with other studies in the specific research area (46, 49, 62, 63). This study showed the practical clinical applicability of GOE as an ingredient that can effectively support the first weeks in smoking reduction or cessation attempts, which are considered to be a critical period for a successful quitting in the long term (64, 65). Further studies should be planned to confirm the benefits of GOE supplementation during smoking reduction or cessation experience, particularly including population of heavy smokers.

## 5 Conclusion

GOE (Neuravena^®^) supplementation provided greater improvements in quality of life measures as well as parameters quantifying stress and sleep during smoking reduction or cessation as compared to those consuming placebo. The beneficial effects were also demonstrated by the higher prevalence of successful smoking reducers at the end of the intervention period in the GEO group. Therefore, Neuravena^®^ may be useful to support subjects in the process of reducing their smoking consumption.

## Data availability statement

The original contributions presented in the study are included in the article/Supplementary material, further inquiries can be directed to the corresponding authors.

## Ethics statement

The studies involving humans were approved by the Ethics Committee of Universidad Católica San Antonio (protocol code CE102004). The studies were conducted in accordance with the local legislation and institutional requirements. The participants provided their written informed consent to participate in this study.

## Author contributions

MF: Conceptualization, Writing – original draft, Writing – review & editing, Data curation. AG-M: Investigation, Writing – original draft, Writing – review & editing, Data curation. AL: Conceptualization, Writing – review & editing. SP-P: Investigation, Writing – review & editing. DV-M: Investigation, Writing – review & editing. FL-R: Conceptualization, Formal analysis, Writing – review & editing. AG-G: Investigation, Writing – review & editing. JM-M: Investigation, Writing – review & editing. FC: Conceptualization, Investigation, Writing – original draft, Writing – review & editing. EI: Conceptualization, Writing – original draft, Writing – review & editing. JJ: Writing – original draft, Writing – review & editing.

## Footnotes

^1^https://www.who.int/tools/whoqol
